# MiR-489-3p inhibits cell proliferation, migration, and invasion, and induces apoptosis, by targeting the BDNF-mediated PI3K/AKT pathway in glioblastoma

**DOI:** 10.1515/biol-2020-0024

**Published:** 2020-05-29

**Authors:** Bo Zheng, Tao Chen

**Affiliations:** Department of Neurosurgery, Jingzhou Central Hospital, Hubei Province, Jingzhou, 434020, China

**Keywords:** apoptosis, brain-derived neurotrophic factor, glioblastoma, invasion, migration, miR-489-3p

## Abstract

Among astrocyte tumors, glioblastoma (GBM) is the most malignant glioma, highly aggressive and invasive, with extremely poor prognosis. Previous research has reported that microRNAs (miRNAs) participate in the progression of many cancers. Thus, this study aimed to explore the role and the underlying mechanisms of microRNA (miR)-489-3p in GBM progression. The expression of miR-489-3p and brain-derived neurotrophic factor (BDNF) mRNA was measured by quantitative real-time polymerase chain reaction. Western blot analysis was used to detect BDNF protein and the PI3K/AKT pathway-related protein. Cell proliferation, apoptosis, migration, and invasion were analyzed using CKK-8 assay, flow cytometry, and transwell assay, respectively. The interaction between BDNF and miR-489-3p was explored by luciferase reporter assay and RNA immunoprecipitation (RIP) assay. MiR-489-3p was down-regulated and BDNF was up-regulated in GBM tissues and cells. MiR-489-3p re-expression or BDNF knockdown inhibited GBM cell proliferation, migration, and invasion, and promoted apoptosis. BDNF was a target of miR-489-3p, and BDNF up-regulation reversed the effects of miR-489-3p on GBM cells. The protein levels of p-AKT and p-PI3K were notably reduced in GBM cells by overexpression of miR-489-3p, but were rescued following BDNF up-regulation. Therefore, miR-489-3p inhibited proliferation, migration, and invasion, and induced apoptosis, by targeting the BDNF-mediated PI3K/AKT pathway in GBM, providing new strategies for clinical treatment of GBM.

## 1 Introduction

Glioblastoma (GBM), accounting for more than 80% of all tumors in the brain, is the most malignant glioma among astrocyte tumors [1]. According to the grading system developed by the World Health Organization (WHO), GBM belongs to grade IV of gliomas, indicating that it is highly aggressive and invasive and associated with extremely poor prognosis [2]. The overall survival time of GBM patients ranges from 12 to 18 months [3], and fewer than 5% of patients survive more than 5 years after diagnosis [4]. Despite the growth in experimental investigations in GBM, there is little significant progress. Therefore, we must understand the mechanism and explore the potential therapeutic targets for GBM.

MicroRNAs (miRNAs) are a class of endogenous, non-coding, single-stranded small RNAs of approximately 21–23 nucleotides in length. Due to their capacity to achieve post-transcriptional silencing of tumor suppressors or oncogenes, miRNAs are regarded as important regulators of gene expression, both in pathological and physiological conditions [5]. In recent years, many miRNA molecules were identified in different GBM samples [6,7], most of them are highly expressed while a few are down-regulated compared with normal tissues; all of them display oncogenic or antioncogenic features though the regulation of crucial biological processes, such as proliferation, metastasis, apoptosis, DNA repair, and so on [8,9,10].

MicroRNA-489-3p (miR-489-3p) is a newly identified miRNA that has been revealed to act as a tumor suppressor in some cancers. For example, Liu et al. discovered miR-489-3p down-regulation promoted osteosarcoma metastasis by activating the PAX3-MET pathway [11]. However, Chen et al. reported that miR-489-3p was up-regulated in renal cell carcinoma (RCC) and promoted chemoresistance to oxaliplatin in RCC cells [12]. Therefore, the role of miR-489-3p depends on the disease type as well as the microenvironment of the disease. In GBM, Zhang et al. reported that long noncoding RNA (lncRNA) LINC01446 promoted GBM progression by regulating TPT1 through miR-489-3p [13], indicating the potential involvement of miR-489-3p in GBM progression. However, the exact role of miR-489-3p in GBM remains unclear. Brain-derived neurotrophic factor (BDNF) is a protein synthesized in the brain and widely distributed throughout the central nervous system. During the development of the central nervous system, it plays an important role in the survival, differentiation, and growth of neurons [14]. Recent studies demonstrated that BDNF participates in the carcinogenesis of glioma [15,16]. Thus, BDNF may be a promising factor in GBM treatment.

In this study, we found that miR-489-3p was down-regulated in GBM tissues and cells. miR-489-3p specifically targeted BDNF and regulated GBM cell proliferation, migration, invasion, and apoptosis, suppressing the BDNF-mediated PI3K/AKT signaling pathway. We first evaluated a new miR-489-3p/BDNF axis signaling pathway in GBM, which provided new insights in the regulation of GBM progression.

## 2 Materials and methods

### 2.1 Patients and specimens

A total of 30 primary GBM tissue and normal brain tissue specimens were obtained from Jingzhou Central Hospital between September 2017 and January 2018, and the clinicopathological parameters of the GBM patients were collected (Table 1). All patients were pathologically diagnosed according to the diagnostic standard of GBM, and none of the patients had received chemo- or radiotherapy prior to the study. All samples were immediately stored at −80°C until required.

**Table 1:** Analysis of the correlation between expression of BDNF and clinicopathological parameters in glioma patients

Variables	Patients, *n*	BDNF expression		*P* value
		Low	High	
Age, years	23	10	13	0.528
<45	10	4	6	
≥45	13	6	7	
Sex				0.632
Male	11	6	5	
Female	12	5	7	
Clinical grading				0.016
I–II	8	6	2	
III–IV	15	4	11	
Tumor size				0.145
≥4.5 cm	11	4	7	
<4.5 cm	12	7	5	


**Informed consent:** Informed consent has been obtained from all individuals included in this study.
**Ethical approval:** The research related to human use has been complied with all the relevant national regulations, institutional policies, and in accordance to the tenets of the Helsinki Declaration. The Ethics Committee of Jingzhou Central Hospital approved this study.

### 2.2 Cell culture

Human GBM cell lines LN229, U251, normal astrocyte cells (NHAs), and HEK-293T cells were obtained from the Cell Bank of the Chinese Academy of Sciences (Shanghai, China). All cells were maintained in Dulbecco’s Modified Eagle Medium (Gibco, Carlsbad, CA, USA) with 10% fetal bovine serum, 100 μg/mL streptomycin, and 100 U/mL penicillin at 37°C with the 5% CO_2_.

### 2.3 Cell transfection

Small interfering RNA (siRNA) targeting BDNF (si-BDNF), siRNA negative control (si-NC), pcDNA, and pcDNA-BDNF overexpression vector (BDNF) were synthesized by GenePharma (Shanghai, China). The miR-489-3p mimic (miR-489-3p, 5′-GUGACAUCACAUAUACGGCAGC-3′) and the corresponding negative control (miR-NC, 5′-UUCUCCGAACGUGUCACGUTT-3′) were purchased from IONEEC (Shanghai, China). Then, 2 μg of synthetic vectors and 10 mM of synthetic oligonucleotides were transfected into LN229 and U251 cells using Lipofectamine 2000 (Invitrogen, Carlsbad, CA, USA) according to the manufacturer’s instructions.

### 2.4 Western blot

Total protein was isolated from the tissues and cells by RIPA lysis buffer (Thermo Scientific™, Waltham, MA, USA) and was quantified using BCA method (Beyotime, Shanghai, China). Fifty microgram protein lysate was subjected by 8% SDS-PAGE gel, transferred onto polyvinylidene fluoride membranes (PVDF; Millipore, MA, USA), and then blocked with 5% nonfat milk for 1 h at room temperature. Subsequently, samples were incubated with primary antibodies against BDNF (1:10,000, ab108319; Abcam, Cambridge, MA, USA), p-PI3K (1:1,000, ab182651; Abcam), PI3K (1:1,000, ab40776; Abcam), p-AKT (1:1,000, 9271; Cell Signaling Technology, Boston, MA, USA), AKT (1:1,000, #9272; Cell Signaling Technology), and β-actin (1:3,000, #4967; Cell Signaling Technology) overnight at 4°C, followed by interaction with HRP-conjugated secondary antibody (1:3,000; ab9482; Abcam) at room temperature for 2 h. Finally, the protein signals were visualized by an enhanced chemiluminescence (Beyotime) and quantitated by Image Lab software (Bio-Rad, Hercules, CA, USA). The β-actin was regarded as an internal control.

### 2.5 Quantitative real-time polymerase chain reaction (qRT-PCR)

Total RNA was isolated from GBM tissues and cells by using TRIzol reagent (Invitrogen). Then, total RNA was reversely transcribed into cDNA via the Exiqon Universal cDNA Synthesis Kit II (Qiagen, Hilden, Germany). After that, qPCR was performed using SYBR Green Real-Time PCR Master Mixes (Qiagen) following the manufacturer’s protocol. U6 and GAPDH were regarded as internal controls. All special primers for U6, miR-489-3p, BDNF, and GAPDH were purchased from Qiagen Company. The relative expression level was analyzed using 2^−ΔΔCt^ method. The sequences of primers used in this study were listed as followed: miR-489-3p: F: 5′-CTGACATGTGAGAGGCACTCAA-3′, R: 5′- GCTGCCGTATATGTGATGTCACT-3′; BDNF: F: 5′-AAGTGCCTTTGGAGCCTCCT-3′, R: 5′-GCTAATACTGTCACACACGC-3′; GAPDH: F: 5′-CGCTCTCTGCTCCTCCTGTTC-3′, R: 5′-ATCCGTTGACTCCGACCTTCAC-3′; U6, F: 5′-CTCGCTTCGGCAGCACA-3′, R: 5′-CGCTTCACGAATTTGCGT-3′.

### 2.6 Cell proliferation, metastasis, and apoptosis

Cell Counting Kit-8 (CCK-8; Beyotimes) was used to determine cell proliferation. LN229 and U251 cells were seeded into a 96-well plate at a density of 3,000/well and cultured at 37°C with 5% CO_2_ for 24 h. Then, per well were interacted with 10 μL CCK-8 solution for another 2 h at 37°C. Finally, the absorbance was measured at 450 nm using a microplate reader (Bio-Rad).

Transwell assays were carried out using chamber inserts precoated with or without Matrigel (BD Biosciences, San Jose, CA, USA) to analyze the migration and invasion of LN229 and U251. Experiments were repeated in triplicate independently. Details of the procedure are based on the literature as described previously [17].

For apoptosis analysis, LN229 and U251 cells were gathered and Annexin V-FITC/PI assay kit (BD Biosciences) was used according to the manufacturer’s protocol. Then, FACScan® flow cytometry (BD Biosciences) was carried out to detect cell apoptotic rate.

### 2.7 Luciferase reporter assay

The wild-type (wt) or mutant (mut) BDNF 3′-UTR harboring miR-489-3p putative target sites were amplified and inserted into pGL3 vectors (Promega, Madison, WI, USA) to generate BDNF-wt or BNDF-mut luciferase reporter vector (pGL3-BDNF-wt or -mut), respectively. Then, 40 nM of miR-489-3p mimic or miR-NC and 100 ng of pGL3-3′UTR-wt or mut were cotransfected into LN229 and U251 cells using Lipofectamine 2000 (Invitrogen). Relative luciferase activity was analyzed using a dual luciferase assay kit (Promega) in each cell group according to the manufacturer’s instructions.

### 2.8 RNA immunoprecipitation

RIP assay was performed using Magna RNA immunoprecipitation kit (Millipore). LN229 and U251 cells transfected with miR-489-3p or miR-NC were lysed in RIP buffer, and then cell lysate was incubated with magnetic beads coated with anti-Ago2 (Abcam) or negative control IgG antibody (Sigma, St Louis, MO, USA) at 4°C. Subsequently, the immunoprecipitated RNA was isolated and detected by qRT-PCR.

### 2.9 Statistical analysis

All data from at least three independent experiments were represented as mean ± SD and analyzed using GraphPad Prism 7 (GraphPad Inc., San Diego, CA, USA). Significant differences were analyzed using Student’s *t*-test or one-way analysis of variance (ANOVA) followed by Dunnett’s test. *P* value less than 0.05 was considered significant.

## 3 Results

### 3.1 BDNF is up-regulated in GBM tissue and cells

**Figure 1:**
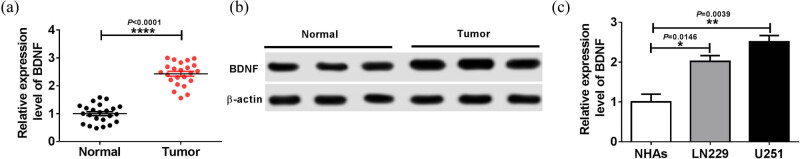
The expression of BDNF in GBM tissues and cells. The expression of BDNF was measured in GBM tissues (a), (b), and cells (c) by western blot and qRT-PCR, respectively. *P < 0.05, **P < 0.01, ***P < 0.001, ****P < 0.0001.

To identify potential effects of BDNF on GBM progression, the expression of BDNF was measured in GBM tissues and cells. The data showed that the protein level of BDNF was significantly elevated in GBM tissue compared with the normal group (Figure 1a and b, *P* < 0.0001), and the mRNA of BDNF in LN229 and U251 cells also was highly expressed compared with the NHAs cells (Figure 1c, *P* = 0.014; *P* = 0.0039). In addition, based on the level of BDNF, GBM patients were divided into two groups, and higher BDNF expression was correlated with clinical grading (*P =* 0.016, Table 1). These data revealed that BDNF expression was dysregulated in GBM, and its high expression was related to the clinical grading of patients with GBM.

### 3.2 Silencing of BDNF inhibits GBM cell proliferation and metastasis, and promotes apoptosis

**Figure 2:**
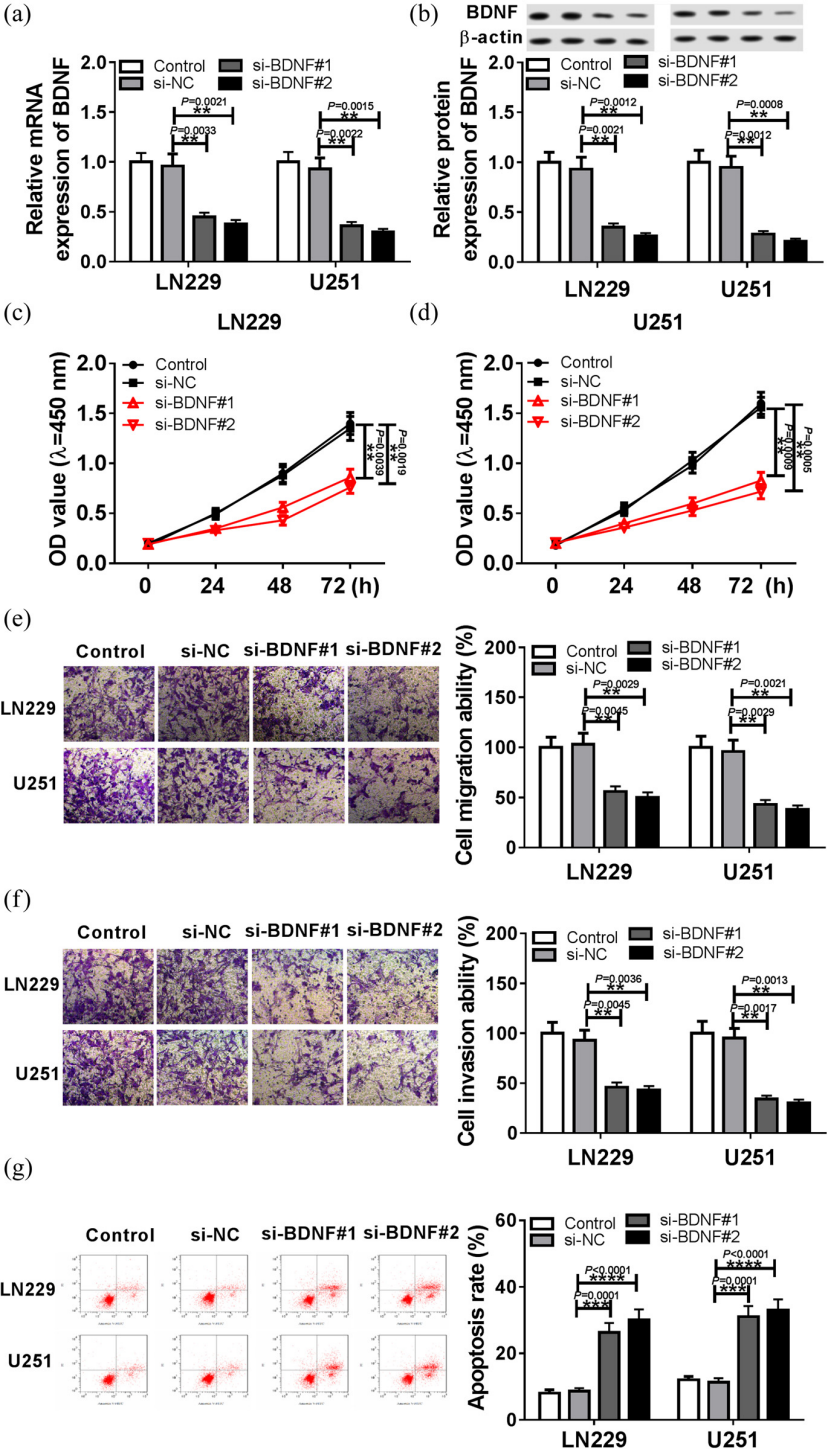
The effects of BDNF on proliferation, metastasis, and apoptosis of GBM cells. LN229 and U251 cells were transfected with si-NC or si-BDNF. The mRNA (a) and protein (b) expression of BDNF were analyzed using qRT-PCR and western blot analysis in LN229 and U251 cells, respectively. The proliferation (c and d), migration (e), invasion (f), and apoptosis (g) in LN229 and U251 cells were detected using CCK-8 assay, transwell assay, and flow cytometry, respectively. *P < 0.05, **P < 0.01, ***P < 0.001, ****P < 0.0001.

BDNF expression was decreased using si-BDNF#1 and si-BDNF#2. BDNF expression was significantly reduced in LN229 and U251 cells transfected with si-BDNF (Figure 2a and b, *P* < 0.001). Subsequently, the role of BDNF on GBM cell proliferation, metastasis, and apoptosis were analyzed. Results illustrated that BDNF knockdown (both si-BDNF#1 and si-BDNF#2) suppressed LN229 and U251 cell proliferation (Figure 2c and d, *P* < 0.001), migration (Figure 2e, *P* < 0.001), and invasion (Figure 2f, *P* < 0.001), while the apoptosis rate was significantly escalated by the silence of BDNF (Figure 2g, *P* < 0.001 in si-BDNF#1, *P* < 0.0001 in si-BDNF#2). Taken together, BDNF silence impacted the progression of GBM.

### 3.3 BDNF is a target of miR-489-3p, and miR-489-3p negatively regulated BDNF expression

**Figure 3:**
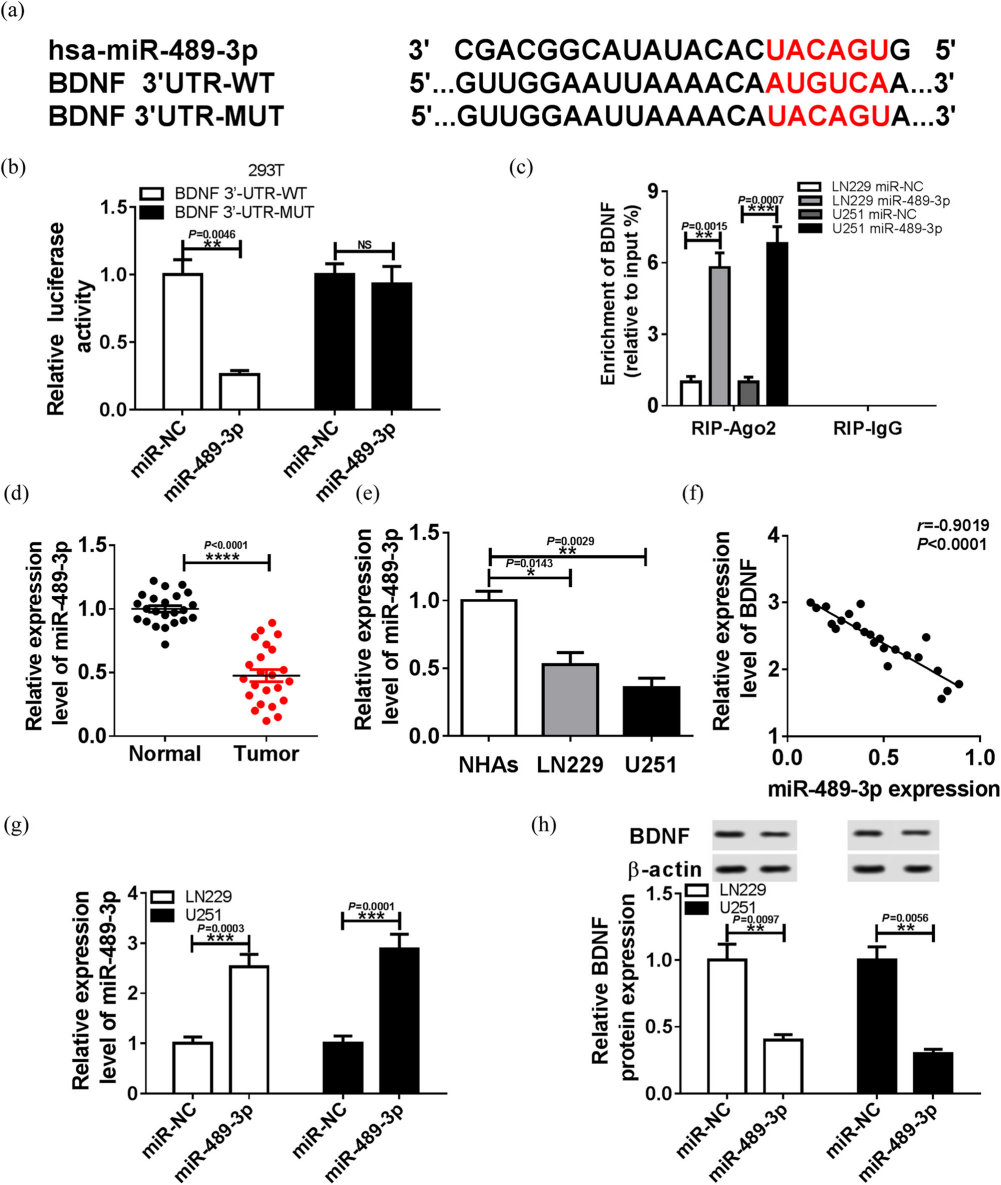
The association between BDNF and miR-489-3p in GBM cells. (a) The putative binding sites of BDNF 3′-UTR and miR-489-3p. (b) Luciferase activity was measured in LN229 and U251 cells co-transfected with BDNF-wt or BDNF-mut and miR-NC or miR-489-3p. (c) The enrichment of BDNF was discovered in GBM cells transfected with miR-NC or miR-489-3p after RIP. The expressions of miR-489-3p were measured in GBM tissues (d) and cells (e) using qRT-PCR. (f) Correlation analysis between miR-489-3p and BDNF. (g) The expression of miR-489-3p in LN229 and U251 cells transfected with miR-NC or miR-489-3p. (h) The protein expression of BDNF was analyzed using the Western blot in LN229 and U251 cells transfected with miR-NC or miR-489-3p. *P < 0.05, **P < 0.01, ***P < 0.001, ****P < 0.0001.

By searching the StarBase database, BDNF was found to be a potential target gene of miR-489-3p. The potential binding sites of miR-489-3p and BDNF are shown in Figure 3a. Immediately, the luciferase reporter assay was performed and results showed luciferase activity was remarkably reduced in 293 T cells co-transfected with BDNF-wt and miR-489-3p compared with the NC control group, while there was no change in the BDNF-mut group (Figure 3b, *P* = 0.0046). RIP assay also indicated that overexpression of miR-489-3p clearly engendered enrichment of BDNF after Ago2 RIP; however, its efficacy was lost in response to IgG RIP in LN229 (*P* = 0.0015) and U251 cells (*P* = 0.0007) (Figure 3c). These data confirm the direct interaction between BDNF and miR-489-3p. After that, miR-489-3p expression in GBM was detected, results indicate that the level of miR-489-3p was significantly decreased in GBM tissues (*P* < 0.0001) and cells (*P* = 0.0143, *P* = 0.0029) compared with the NC group (Figure 3d and e), and BDNF expression was negatively correlated with miR-489-3p in GBM tissue (*r* = −0.9019; *P* < 0.0001) (Figure 3f). Furthermore, LN229 and U251 cells were transfected with miR-NC or miR-489-3p, and the levels of miR-489-3p were significantly increased in LN229 (*P* = 0.0003) and U251 (*P* = 0.0001) cells. Western blot analysis showed that miR-489-3p overexpression inhibited the level of BDNF LN229 (*P* = 0.0097) and U251 (*P* = 0.0056) cells (Figure 3g and h). All the above results indicate that miR-489-3p specifically suppressed BDNF expression.

### 3.4 BDNF reverses the anti-tumor effects of miR-489-3p on GBM cell progression

**Figure 4:**
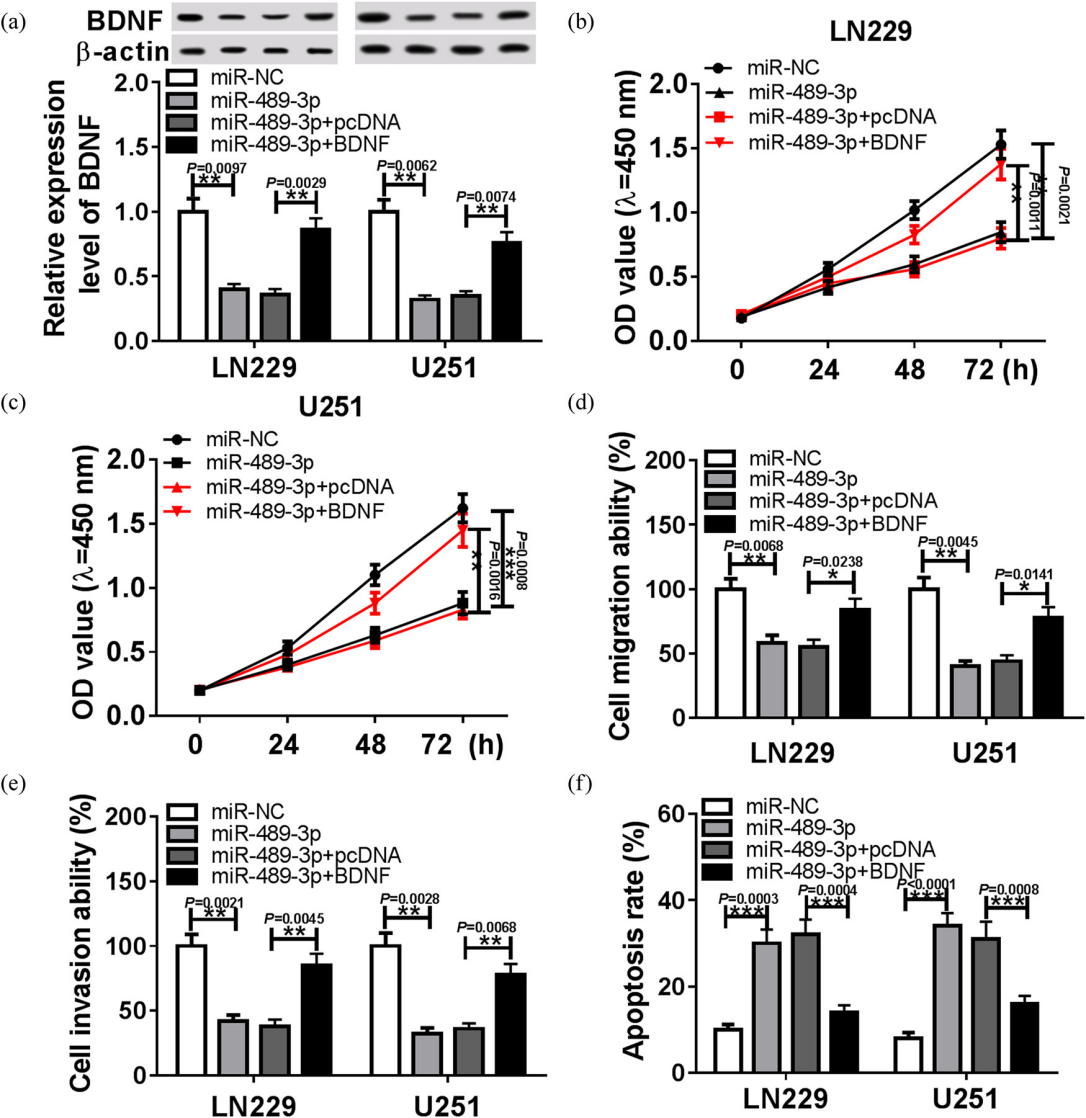
The regulatory effect of miR-489-3p on BNDF-mediated GBM cells proliferation, metastasis, and apoptosis. LN229 and U251 cells were transfected with miR-NA, miR-489-3p, miR-489-3p + pcDNA, or miR-489-3p and BDNF. (a) The protein expression of BDNF was analyzed using the Western blot after transfection. The proliferation (b and c), migration (d), invasion (e), and apoptosis (f) of LN229 and U251 cells were detected using CCK-8 assay, transwell assay, and flow cytometry, respectively. *P < 0.05, **P < 0.01, ***P < 0.001, ****P < 0.0001.

Cells were transfected with miR-NC, miR-489-3p, miR-489-3p + pcDNA, or miR-489-3p + BDNF. We found that miR-489-3p overexpression inhibited the level of BDNF, while this inhibition was rescued by BDNF up-regulation, indicating the successful transfection (Figure 4a, *P* < 0.001). Afterward, rescue assay was performed, and results demonstrated miR-489-3p overexpression inhibited LN229 and U251 cell proliferation (Figure 4b, C, *P* < 0.01), migration (Figure 4d, *P* < 0.05), invasion (Figure 4e, *P* < 0.01), and induced apoptosis (Figure 4f, *P* < 0.0001), whereas these anticancer effects were attenuated by BDNF up-regulation (Figure 4b–f). Therefore, we concluded miR-489-3p could inhibit GBM cell progression via regulating BDNF.

### 3.5 MiR-489-3p inhibits PI3K/AKT signaling in GBM cells via BDNF

**Figure 5:**
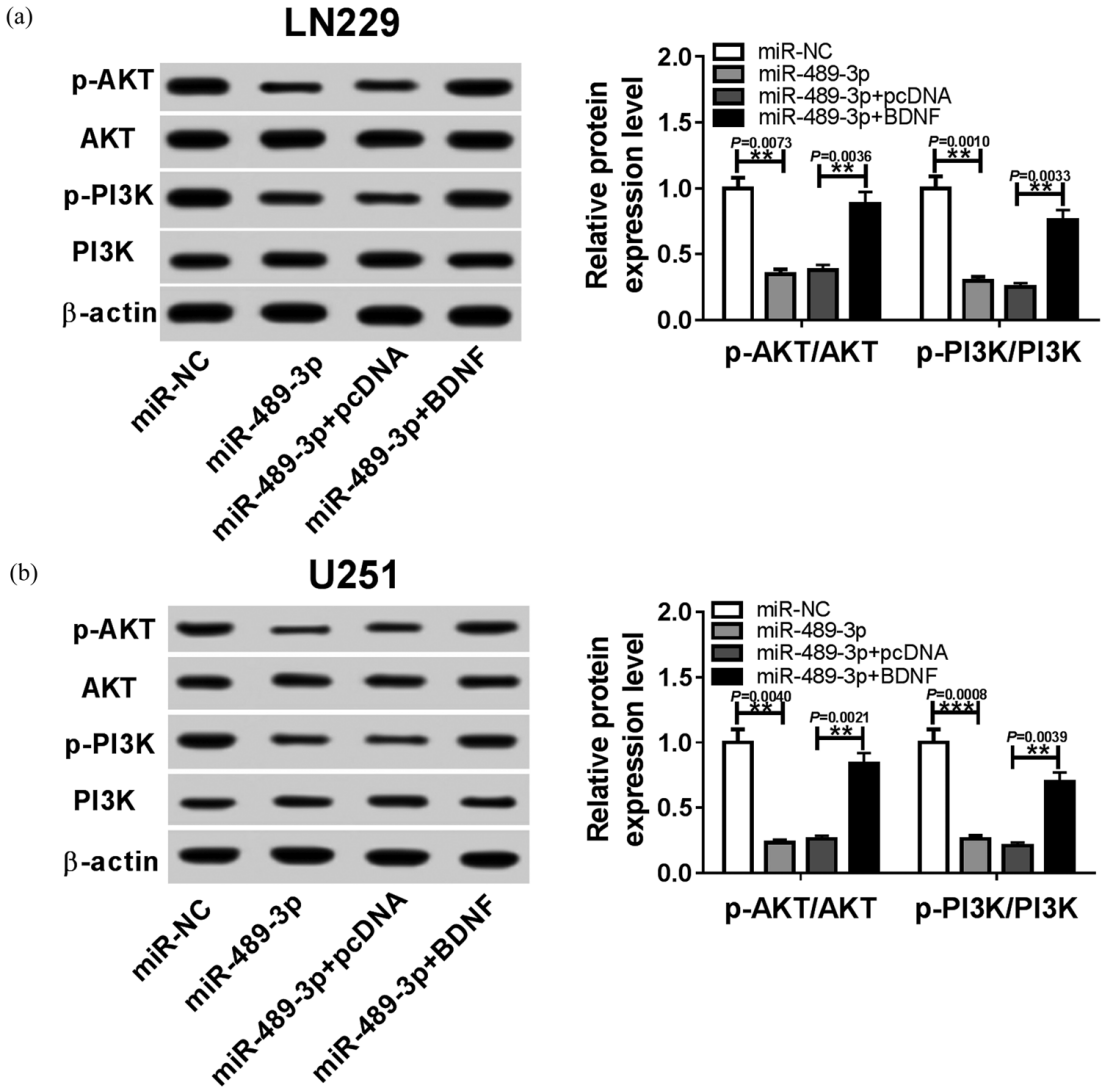
MiR-489-3p regulates PI3K/AKT signaling pathway via BDNF. The protein expression of p-AKT, AKT, p-PI3K, and PI3K were analyzed using the Western blot after transfection with miR-NA, miR-489-3p, miR-489-3p + pcDNA, or miR-489-3p + BDNF in LN229 (a) and U251 (b) cells, respectively. *P < 0.05, **P < 0.01, ***P < 0.001, ****P < 0.0001.

BDNF has been revealed to be involved in the regulation of the PI3K/AKT signaling pathway [18,19,20]. Thus, we further investigated whether miR-489-3p could activate the PI3K/AKT pathway. The proteins of p-AKT, AKT, p-PI3K, and PI3K were detected using western blot in LN229 and U251 cells. Subsequently, results indicated the protein levels of p-AKT and p-PI3K were notably reduced by miR-489-3p overexpression, but restored by following BDNF up-regulation; there were no changes in the protein levels of AKT and PI3K in LN229 (*P* < 0.01) and U251 cells (*P* < 0.01) (Figure 5a and b). Altogether, PI3K/AKT signaling pathway might be regulated by miR-489-3p via BDNF.

## 4 Discussion

Currently, biological molecular therapies have been identified in diverse tumors, and multiple biomarkers and biological targets have been found [21]. In glioma, promising strategies of biological molecular therapies have been worked out, especially for these high-grade glioma patients [21,22]. Growing evidence has validated the possibility of miRNAs as candidate therapeutic targets by involvement in various physical and pathological processes that regulate the development and progression of glioma [7,8,9]. For example, miR-34a hinders cell progression and chemoresistance via targeting PD-L1 in glioma [23]. MiR-429 suppresses glioma invasion through the inhibition of BMK1, both *in vitro* and *in vivo* [24]. More recently, miR-489-3p has been proposed to be associated with the progression of GBM and might be a potential therapeutic target for glioma [13]. Therefore, we focused on the role of miR-489-3p in GBM. In this study, we demonstrated that miR-489-3p is significantly decreased in GBM tissues and cells, and up-regulating miR-489-3p inhibits GBM progression by suppressing cell proliferation, migration and invasion, and by promoting cell apoptosis.

A previous study has identified miR-489-3p targeted TPT1 in GBM cells [13]. As each miRNA can have multiple target genes, other target genes of miR-489-3p may also play important roles in GBM. Potential miRNA targets of miR-489-3p were therefore predicted, and BDNF was validated as a target of miR-489-3p. BDNF has been reported to be elevated and involved in the tumorigenesis and progression of various human cancer types, including cancers of the lung, stomach, breast, and cervix, as well as hepatocellular carcinoma and chondrosarcoma [25,26,27,28,29,30]. In glioma, mature BDNF was also up-regulated [31]. Liu et al. found miR-210 inhibited cell migration and invasion by targeting BDNF in GBM [16]. MiR-103 suppressed glioma cell proliferation and invasion by targeting BDNF [17]. These studies suggest that BDNF is valuable for investigation as a potential target for the treatment of GBM patients. In this study, BDNF was increased in GBM tissues and cells, and BDNF knockdown suppressed GBM cell tumorigenesis. Furthermore, rescue experiments demonstrated that BDNF overexpression essentially reversed the anti-tumor effects mediated by miR-489-3p re-expression on GBM cells. Thus, we have evaluated a new miR-489-3p/BDNF axis signaling pathway in GBM, which could provide new insight in regulating GBM progression.

BDNF has been confirmed to be involved in PI3K/AKT signaling pathway regulation [18–20]. Binding of BDNF to its major receptor, tropomyosin-related receptor kinase B (TrkB), has high affinity and specificity, which causes the activation of downstream signaling pathway PI3K/AKT [32]. In this study, we found that the protein levels of p-AKT and p-PI3K were notably reduced in GBM cells by miR-489-3p re-expression, but were enhanced by BDNF overexpression, thus, miR-489-3p suppressed the activation of PI3K/AKT signaling pathway by BDNF suppression. All these results suggest that miR-489-3p might exert anti-tumor effects in GBM by inactivating the PI3K/AKT signaling pathway through BDNF.

In conclusion, our findings demonstrated that miR-489-3p inhibits GBM cell proliferation, migration, and invasion, and induce apoptosis, by regulating the BDNF-mediated PI3K/AKT signaling pathway, providing new potential gene-targeting strategies for the treatment of GBM.
